# The Core Circadian Clock Gene *PiLUX*, a MYB-Family Transcription Factor, Enhances Cold Tolerance in *Plumbago indica* L.

**DOI:** 10.3390/plants15193032

**Published:** 2026-10-03

**Authors:** Zi-An Zhao, Yi-Rui Li, De-Hui Li, Hai-Jun Liao, Ting-Jiang Gan

**Affiliations:** 1 Engineering Research Centre of Chuanxibei Rural Human Settlement (RHS) Construction, Mianyang Teachers’ College, Mianyang 621016, China; 2 College of Life Sciences Agri-Forestry, Southwest University of Science and Technology, Mianyang 621010, China; 3 School of Urban and Rural Planning and Construction, Mianyang Teachers’ College, Mianyang 621016, China

**Keywords:** *Plumbago indica* L., low-temperature stress, *PiLUX*, cold tolerance, CBF pathway

## Abstract

Low-temperature stress is a key environmental factor limiting the introduction and cultivation of plants native to tropical and subtropical regions; however, the molecular mechanisms governing their cold tolerance are not fully understood. In this study, we identified a core circadian rhythm gene—*PiLUX*—in the ornamental and medicinal plant *Plumbago indica* L. (*P. indica*) and systematically analyzed its role in the plant’s response to low temperatures. Transcriptomic analysis and physiological measurements indicated that *PiLUX* is significantly upregulated following cold induction; furthermore, its transcriptional levels show positive correlations with cold-tolerance-related physiological indicators such as reactive oxygen species (ROS) scavenging capacity, osmoregulatory substance accumulation, and membrane lipid peroxidation. In transgenic *Arabidopsis thaliana* (*A. thaliana*), heterologous overexpression of *PiLUX* enhances cold tolerance but also delays flowering, suggesting that reproductive development is sacrificed to conserve nutrients for cold tolerance. VIGS experiments confirmed that *PiLUX* knockdown exacerbates oxidative damage in *P. indica* under cold stress and reduces its recovery ability. The experimental results and transcriptomic data suggest that *PiLUX* may participate in the low-temperature response downstream of, or in parallel with, the CBF pathway; however, direct regulatory evidence is still required. This study provides insights into the adaptation strategies of thermophilic plants under low-temperature stress and offers a theoretical basis and genetic resources for cold-tolerance breeding in *P. indica*.

## 1. Introduction

Low-temperature stress is an environmental factor that significantly affects plant growth, productivity, and survival and limits the geographic distribution of plant species [1,2]. Most temperate plants can adapt to low temperatures—a process known as cold acclimation—and exhibit improved cold tolerance after exposure to non-freezing low temperatures [3,4]; in contrast, plants in tropical and subtropical regions lack cold acclimation mechanisms, are unable to overwinter outdoors, and face survival challenges under low-temperature stress [5,6]. Worldwide, economic losses from cold-related disasters to crops and horticultural plants have been estimated to reach up to USD 2 billion annually [7]. For plants native to tropical or subtropical regions, a lack of cold acclimation means they face severe challenges from low winter temperatures when introduced and cultivated in temperate regions, which severely restricts the promotion and application of excellent germplasm [8,9]. Therefore, elucidating the mechanisms underlying plants’ responses to low temperatures and identifying key cold-tolerance genes are of great significance for expanding the cultivation range of plant species and ensuring the stability of agricultural production and landscape gardening.

Low temperatures inhibit plant growth and development, resulting in slow growth, wilted leaves, and impaired flowering and fruit set [10,11]. At the cellular level, low temperatures cause damage to plant cell membranes. Membrane lipids consist primarily of saturated and unsaturated fatty acids; when saturated fatty acids solidify under cold temperatures, they impair membrane fluidity [12,13]. Therefore, thermophilic plants with a higher proportion of saturated fatty acids in their cell membranes suffer even greater damage when exposed to low temperatures [14]. Low-temperature conditions lead to reduced cell membrane fluidity, decreased selective permeability, and dysfunction of membrane proteins, among other adverse effects; at the same time, they cause cold-sensitive symptoms such as electrolyte leakage, insufficient energy supply, and oxidative stress [15,16]. Additionally, low-temperature conditions weaken the ROS scavenging system, leading to a significant accumulation of ROS such as superoxide anions (O^2−^) and hydrogen peroxide (H_2_O_2_), which in turn triggers membrane lipid peroxidation and the production of harmful substances such as malondialdehyde (MDA), resulting in membrane damage [17,18]. Furthermore, under low-temperature stress, plants regulate the expression or activity of specific transcription factors, thereby altering plant hormone metabolism and leading to an imbalance in endogenous hormones and metabolic disorders [19,20].

To cope with damage induced by low temperatures, plants have developed sophisticated mechanisms for cold acclimation over the course of their long evolutionary history; amongst these, the CBF-COR cascade is highly conserved in a variety of plants, including *A. thaliana*, rice (*Oryza sativa*), and wheat (*Triticum aestivum* L.) [20,21,22]. When exposed to low temperatures, plants activate the expression of genes in the CBF family. These genes are members of the AP2/ERF family of transcription factors and recognize the CRT/DRE elements in the *COR* gene promoters; their binding to these promoters activates downstream cold-response genes, which in turn enhance cell membrane stability, promote the scavenging of reactive oxygen species, and mitigate cold damage. In Plumbago plants, heterologous expression of *PaDREB1A* (i.e., *CBF3*) significantly enhances the cold tolerance of transgenic *A. thaliana*, indicating that the CBF-dependent pathway also plays a central role in this genus [23].

Recent studies have shown that plants’ responses to low temperatures are not constant but are strictly regulated by circadian rhythms [10,24,25]. The expression of *CBF1*, *CBF2*, and *CBF3* exhibits circadian fluctuations throughout the day and is more significantly induced by low temperatures at night [8,26]. The core oscillator of the circadian clock consists of multiple nested transcriptional–translational feedback loops, among which the Evening Complex (EC)—composed of LUX ARRHYTHMO (LUX), ELF3, and ELF4—plays a key role in regulating nocturnal gene expression and photoperiodic responses [27,28,29]. It is worth noting that *LUX* contains a conserved MYB domain that is directly involved in the transcriptional regulation of plant growth, development, and defense [30,31]. In *A. thaliana*, the *Atlux* mutation not only reduces the plant’s tolerance to low temperatures but also causes it to bolt and flower prematurely [32,33]. In rice, overexpression of *OsLUX* enhances ROS scavenging capacity and increases tolerance to low temperatures; conversely, knocking out the *oslux* gene reduces rice tolerance to low temperatures [34]. In tea plants (*Camellia sinensis*) and transgenic *A. thaliana*, *CsLUX* enhances tolerance to low temperatures; furthermore, in *CsLUX*-overexpressing plants, members of the *COR* gene family—including *COR15A*, *COR15B*, and *COR414*—were found to be significantly upregulated, suggesting that these genes may be regulated by the upstream *LUX* factor [35]. Low-temperature stress significantly affects the amplitude of the transcriptional cycles of most circadian-related genes, except for *LUX* genes, suggesting that *LUX* may be a key hub gene in the plant response to low temperatures [33,36,37,38].

*P. indica* is a perennial herb native to tropical and subtropical regions that belongs to the genus Plumbago in the family Plumbaginaceae. It is widely cultivated as an ornamental plant for its distinctive long flower stalks, vibrant purplish-red inflorescences, and relatively long blooming period, and plays an important role in landscaping in tropical and subtropical regions. In addition, plumbagin, a compound produced by its root system, exhibits various pharmacological activities, including antibacterial, anti-inflammatory, and antitumor effects, and is considered a natural medicinal compound with significant development potential [39,40]. However, this plant species is highly sensitive to low temperatures, and winter cold snaps often cause leaf wilting and frost damage to floral organs, severely limiting its expansion into open-field cultivation in high-latitude regions [5,11]. Therefore, identifying the genes that regulate cold tolerance in *P. indica* and elucidating the molecular mechanisms underlying its response to low temperatures have become key prerequisites for promoting its cold-tolerant breeding.

In this study, we focused on the gene *PiLUX* in *P. indica*. By combining transcriptomic analysis with functional validation, we aimed to clarify the role of *PiLUX* in the low-temperature response of this species. Transcriptomic data suggest that *PiLUX* is a candidate node connecting circadian regulation and low-temperature signaling, although direct evidence for its integration into the CBF regulatory network remains to be obtained. This study further clarifies the cold-tolerance function of the *LUX* gene and reveals the physiological and molecular mechanisms by which core circadian transcription factors integrate low-temperature signals, thereby providing a theoretical basis and genetic resources for improving the cold tolerance of crops and horticultural plants in the context of climate change.

## 2. Materials and Methods

### 2.1. Plant Materials and Low-Temperature Stress Treatment

The plant materials used in this study included wild-type *P. indica*, *Nicotiana benthamiana* (*N. benthamiana*), and the Colombian ecotype of *A. thaliana*. Among these, the hydroponically grown *P. indica* seedlings were derived from an aseptic tissue culture and cultivated in a 1/2 Hoagland nutrient solution. All materials were placed in a light-controlled incubator for 14-day preculture under the following conditions: a 16 h/8 h light cycle (day/night), a temperature of 23 °C/20 °C, and a light intensity of 8000 lx. During the cultivation period, standard water and fertilizer management practices were uniformly applied, and the containers were rotated clockwise each week to mitigate uneven exposure to light and temperature caused by placement differences.

Transcriptome sequencing samples were collected from the 3rd to 5th young leaves of wild-type *P. indica*, counting from the leaf tip, and were harvested at 0, 4, 8, 12, and 24 h after a low-temperature treatment at 4 °C. Nine plants were selected at each time point, with three biological replicates, following the method described by Zhao et al. [6]. For morphological observations and physiological and biochemical assays, 4-week-old wild-type and overexpressing *A. thaliana* plants were treated at −6 °C for 8 h and then transferred to preculture conditions for 72 h of recovery. Each treatment group consisted of 27 plants with three biological replicates, following the procedure described by Li et al. [23]. In the VIGS interference experiment, four-week-old hydroponically grown, tissue-cultured *P. indica* seedlings were infected and transformed, and then subjected to a 2-day dark treatment. These seedlings were subsequently subjected to cold-temperature treatment at 4 °C for 0, 24, 48, or 72 h, and then transferred to a pre-cultivation environment for 72 h of recovery. Thirty plants were used per treatment, and the six plants with the highest interference efficiency were selected from each group for morphological observation, measurement of physiological and biochemical indicators, and q-PCR validation. In each of the above experiments, samples were collected at specified time points, immediately flash-frozen in liquid nitrogen, and stored at −80 °C for future use.

### 2.2. RNA Extraction and qRT-PCR Analysis

Total RNA was extracted from *P. indica* samples using the RNAprep Pure Plant Kit (TIANGEN, Beijing, China). cDNA was synthesized via reverse transcription in accordance with the instructions for the Evo M-M LV Plus cDNA Synthesis Kit (Accurate Biology, Changsha, China). Quantitative real-time PCR (qRT-PCR) analysis was performed using the iCycler iQ5 system (Bio-Rad, Hercules, CA, USA) and SYBR Green PCR Master Mix (Transgene). Each reaction contained 5 μL of 2× QuantiFast SYBR Green PCR Master Mix (SYBR Green Pro Taq HS qPCR Kit, Accurate Biology, Changsha, China), following the specific method described by Li et al. [5]. All primers were designed and synthesized by Shanghai Geneworks (Shanghai, China, https://www.sangon.com).

### 2.3. Full-Length Cloning of PiLUX, Sequence Analysis, and Vector Construction

Using the CDS sequences obtained from transcriptomic sequencing as the reference sequences, specific primers for cloning the full-length sequence of the target gene *PiLUX* were designed using Premier 5.0, with the forward primer (5′–3′) being ATGGGAGAGGAGGTCCACATG and the reverse primer (5′–3′) being CTATTTTTCACCCGCTGAAAGATG. Sequence analysis was performed using the DNAMAN version 6.0 and the ExPASy online prediction tool (https://web.expasy.org/protparam/) to construct a phylogenetic tree and perform multiple sequence alignments.

The overexpression vector used was the BG-Plant-GFP constitutive overexpression vector, which contains a 35S promoter with a GFP tag (the tag is located at the 3′ end) (Figure S1); the vector bacterial culture was supplied by Chongqing Baoguang Biotechnology Co., Ltd. (Chongqing, China). Sma I was used as the single restriction site, and the high-fidelity (HF) restriction endonuclease Sma I from New England Biolabs was employed to linearize the vector. The TRV-RNA1 and TRV-RNA2 vectors used for VIGS were provided by the Cotton Research Center of the Shandong Academy of Sciences [41]. These vectors were linearized using the high-fidelity (HF) restriction endonucleases EcoR I and Kpn I from New England Biolabs. The sequencing results following insertion into the *PiLUX* vector are provided in the Supplementary Files (Figures S2 and S3).

### 2.4. RNA Extraction, RNA Sequencing (RNA-Seq), Library Construction, and Gene Annotation

The transcriptome data analyzed in this study were obtained from our previously published dataset [6], which has been deposited in NCBI under BioProject accession number PRJNA1172344 (https://www.ncbi.nlm.nih.gov/bioproject/PRJNA1172344, accessed on 10 November 2025). Young leaves of *P. indica* were sampled at 4 °C under cold stress at 0, 4, 8, 12, and 24 h. Total RNA was extracted from *P. indica* using the RNAprep Pure Plant Kit (TIANGEN, China) according to the manufacturer’s instructions. RNA purity was confirmed by 1% agarose gel electrophoresis. The Qubit 2.0 and Agilent 2100 systems were used to analyse the library and ensure the reliability of the results. Complementary DNA (cDNA) libraries were sequenced on the Illumina NovaSeq high-throughput sequencing platform (Illumina, Inc., San Diego, CA, USA), which is based on sequencing by synthesis (SBS) technology. The unigene sequences were compared with those in public databases, including the non-redundant database (NR), Swiss-Prot, Gene Ontology (GO), Clusters of Orthologous Groups (COG), euKaryotic Orthologous Groups (KOG), eggNOG4.5, and Kyoto Encyclopedia of Genes and Genomes (KEGG) databases, using the BLASTx algorithm (NCBI BLAST+ v2.12.0). After the amino acid sequences of the unigenes were predicted, their annotation information was obtained using HMMER v3.4 software and compared with the Pfam database. InterProScan was used to analyse the GO Orthology results of the new genes via the InterPro-integrated databases, KOBAS was used to obtain the KEGG Orthology results of the unigenes in KEGG, and HMMER v3.4 software was used to compare the amino acid sequences of the predicted completed unigenes with those in the Pfam database to obtain annotation information for the unigenes. GO functional annotation and KEGG pathway enrichment analysis were performed using a hypergeometric test, and pathways with *p* < 0.05 were considered significantly enriched.

### 2.5. Subcellular Localization Analysis

The *Agrobacterium tumefaciens* strain GV3101 was used for the transient infiltration of *N. benthamiana* leaves in the subcellular localization experiment. The Agrobacterium suspension, which carries the *PiLUX* overexpression vector, was prepared as described in Section 2.3 and injected into vigorously growing *N. benthamiana* plants that had smooth, tender leaves. Before infection, a sterile needle was used to make 2–4 small punctures on the underside of the leaf, and the bacterial suspension was injected into the leaf tissue through these punctures. After injection, the edges of the infection site were outlined with a marker pen to facilitate precise sampling during subsequent subcellular localization. The infected tobacco plants were cultured in the dark for 36 h, after which the subcellular localization of the target gene was observed using a laser confocal microscope (Olympus FV4000, Evident Corporation, Tokyo, Japan). Images were acquired using the CellSens FV Ver 3.1.1 software, with the scale bar set at 20 μm.

### 2.6. Plant Overexpression Transformation and VIGS Interference

The *Agrobacterium tumefaciens* strain GV3101 was used for both the *A. thaliana* transformation and the VIGS experiments. Any existing pods and open inflorescences were removed from the *A. thaliana* plants prior to transformation. The aerial parts of the plants were immersed in the resuspended bacterial suspension for 1 min, incubated in the dark for 24 h, and then transferred to normal light conditions for a 7-day recovery period. The inoculum was prepared by resuspending the inoculation solution containing 5% sucrose and 0.02% Silwet L-77 (Sigma-Aldrich, St. Louis, MO, USA, CAS: 2478-38-8), and adjusting the OD_600_ to 1.3 ± 0.1. The plants were inoculated three times before being bagged for seed collection. Transformed progeny were screened for resistance using 600 mg/L kanamycin, and qRT-PCR was used to identify positive cases.

VIGS-mediated silencing of *PiLUX* was carried out as described by Lu et al. [41], with modifications. The modifications included the following: (i) the *Agrobacterium tumefaciens* strain GV3101 was used; (ii)the OD_600_ was adjusted to 1.2–1.3; (iii) the mixed bacterial suspension was kept in the dark at room temperature for 6 h; and (iv) inoculation was performed on hydroponically grown *P. indica* seedlings and repeated three times. The detailed procedure was as follows: *CLA1* was used as the positive control. The bacterial suspensions RNA2, which carry the target *PiLUX/CLA1* gene fragment, and RNA1 were mixed with the LB liquid medium (containing 0.1% kanamycin, 0.1% rifampicin, 0.02% acetosyringone, and 0.01% MES) in a 1:9 ratio and incubated in the dark for 12 h. After incubation, bacterial cells were harvested via centrifugation, resuspended in the inoculation solution, and adjusted to an OD_600_ of 1.2–1.3. Subsequently, the RNA1 bacterial suspension was mixed with the RNA2 bacterial suspension containing the *CLA1* or *PiLUX* fragment in a 1:1 ratio and left to stand in the dark at room temperature for 6 h. Vigorous hydroponically grown *P. indica* seedlings were selected for leaf inoculation. Following inoculation, the plants were placed in a light-controlled incubator at 20 °C and kept in the dark for 2 days; the inoculation was repeated three times in total. At 7–10 days post-infiltration, the leaves of *CLA1*-positive control plants were examined for the appearance of photobleaching spots. The presence of these visible silencing markers confirmed the functionality of the VIGS system, allowing the experiment to proceed to subsequent analyses.

The primers for identifying positive *A. thaliana*, the primers for quantifying the *PiLUX* gene in positive plants, and the VIGS primers for *PiLUX* are shown in Table S1.

### 2.7. Measurement of Physiological and Biochemical Parameters

The measurement of MDA, soluble sugars, H_2_O_2_, and O^2−^ was carried out in accordance with the instructions of the respective test kits, which were purchased from the Nanjing Jiancheng Institute of Biological Engineering; the kit reference numbers were A003-1 (MDA), A145-1-1 (soluble sugars), A064 (H_2_O_2_), and A052-1-1 (O^2−^). The third to fifth leaves from the tips of plant shoots were used for analysis. Three biological replicates were set up for each sample, and each experiment was repeated three times.

### 2.8. Data Analysis

The data of representative experiments were analyzed using IBM SPSS 27 and plotted using Origin 2020. The values are presented as means ± standard deviations (SDs). Statistical differences were compared via one-way analysis of variance (ANOVA) followed by the least significant difference (LSD) multiple range test, with the significance levels set as *p* < 0.05 (*), *p* < 0.01 (**), and *p* < 0.001 (***), or analyzed using Tukey’s test when *p* = 0.05.

## 3. Results

### 3.1. Transcriptome Screening Identifies the Circadian Rhythm Pathway and PiLUX as Candidates for Cold Responses in P. indica

**Figure 1:**
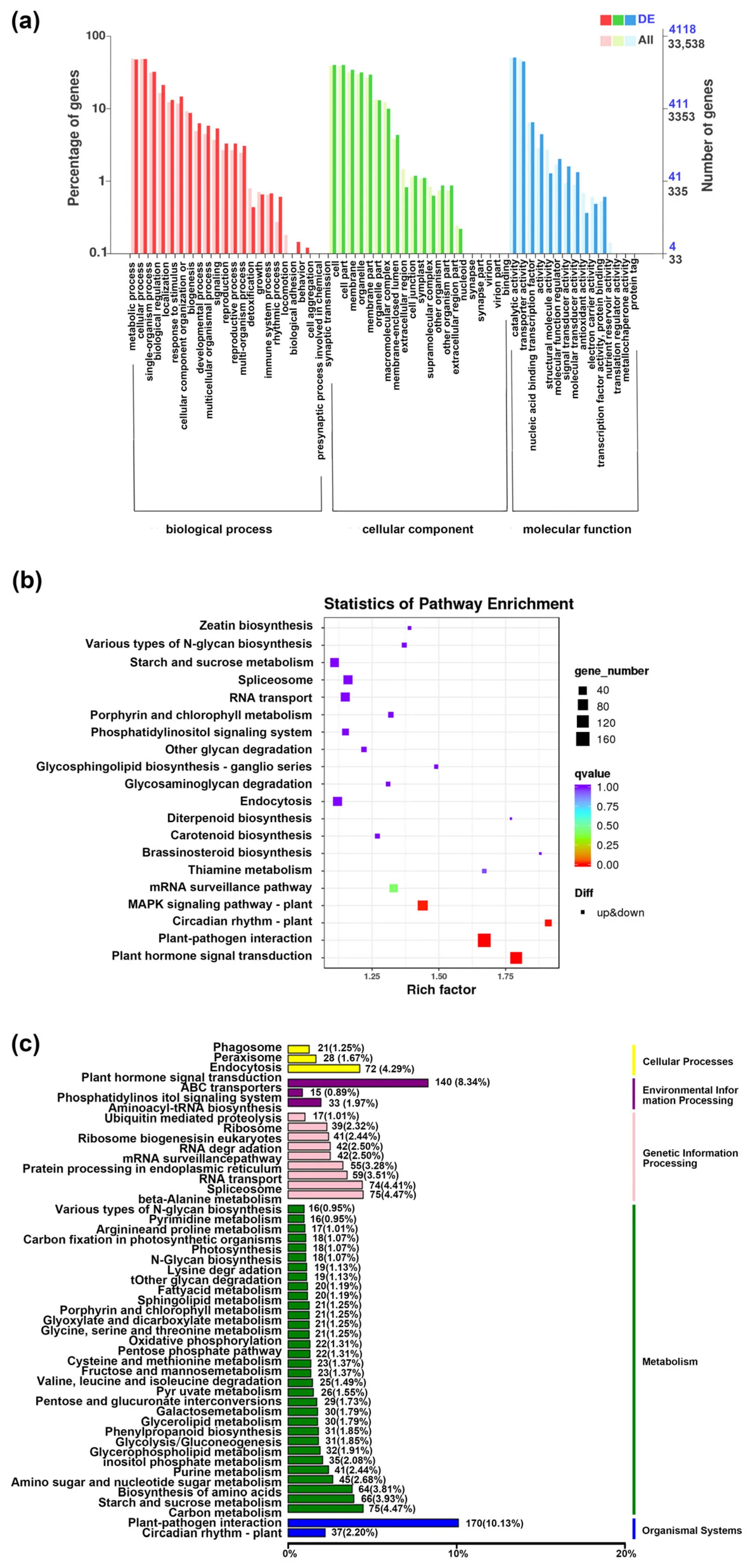
GO functional annotation and KEGG pathway enrichment analysis of differentially expressed genes. (**a**) GO functional annotation; (**b**) bubble plot of KEGG-enriched pathways; (**c**) statistics of differentially expressed genes in significantly enriched KEGG pathways.

To identify candidate genes involved in the low-temperature response of *P. indica*, differentially expressed genes were screened using the thresholds FDR < 0.01 and |log_2_FC| > 1.2, yielding a total of 33,538 unigenes for functional annotation. GO functional annotation and KEGG pathway enrichment analyses were performed on the annotated genes (Figure 1). The KEGG enrichment analysis identified the ‘circadian rhythm–plant’ pathway as one of the significantly enriched pathways (Figure 1b,c). These GO and KEGG results are predictive and were used only to identify candidate pathways and genes; they do not by themselves demonstrate the function of any specific gene in cold tolerance. Based on these results, *PiLUX* was selected as a candidate gene for subsequent experimental validation.

### 3.2. Expression Patterns of the PiLUX Gene in P. indica Under Low Temperatures and Physiological Implications

#### 3.2.1. Analysis of PiLUX Expression Under Low Temperatures

**Figure 2:**
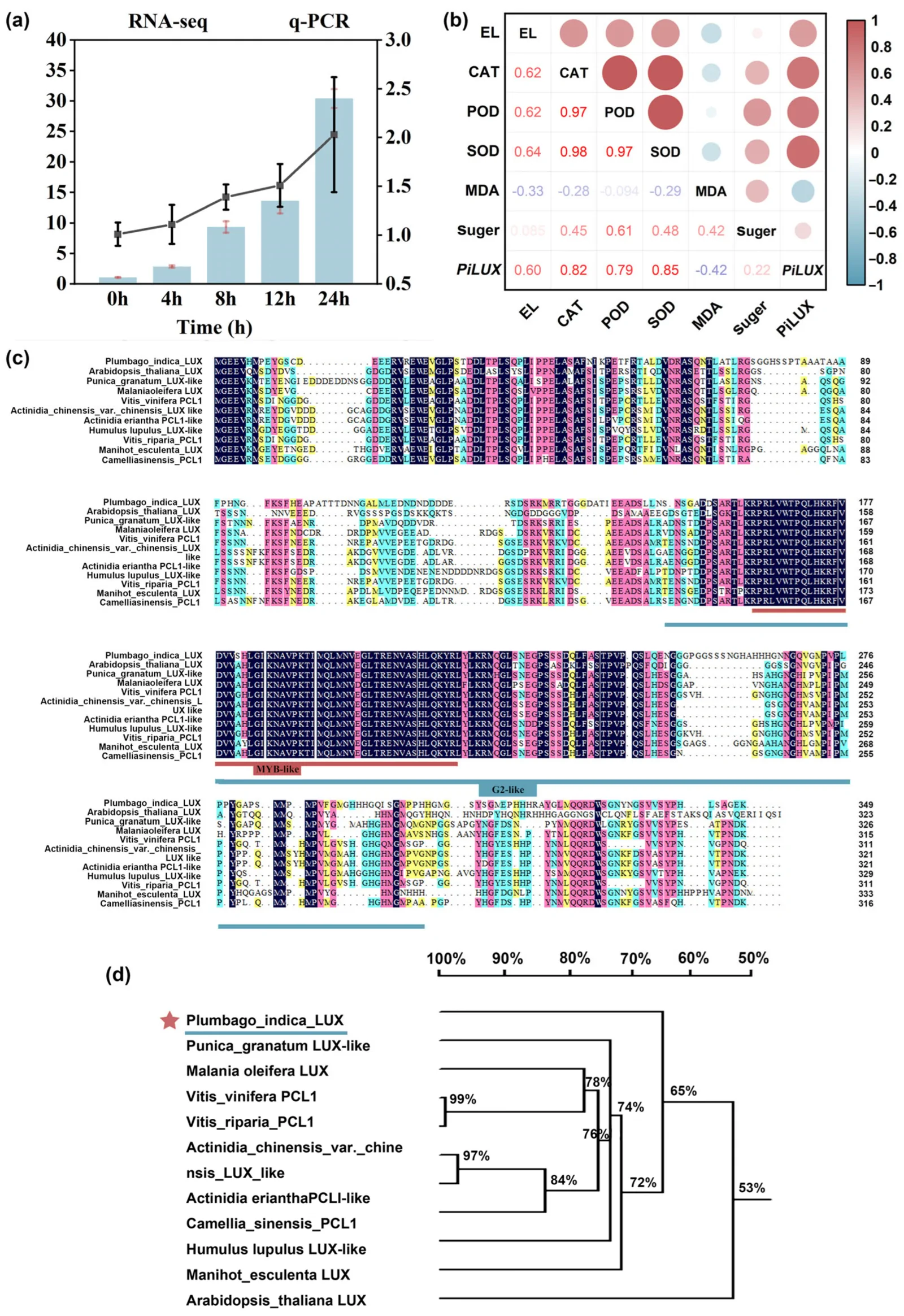
Identification, expression pattern of *PiLUX*, and its correlation with physiological indicators in *P. indica* under low-temperature stress. (**a**) The *X*-axis represents the time of low-temperature treatment, the left *Y*-axis displays the corresponding RNA-seq expression data (blue histogram), and the right *Y*-axis shows the relative gene expression level by qRT-PCR (black dash line). (**b**) Correlation heatmap between *PiLUX* expression and physiological parameters (EL, SOD, POD, CAT, MDA, and soluble sugar) in leaves. The size and color of the circles represent Pearson’s correlation coefficients, with larger and darker red circles indicating stronger positive correlations and larger and darker blue circles indicating stronger negative correlations. Pearson’s correlation coefficients are shown, and the significance of each correlation was assessed by a two-tailed Student’s *t*-test. *p* < 0.05 was considered significant. (**c**) Sequence comparison of PiLUX and homologous proteins of different species. The asterisk indicates the target protein PiLUX. (**d**) Phylogenetic tree of PiLUX constructed using the neighbor-joining method.

To validate the transcriptome FPKM values, the relative expression level of *PiLUX* under low-temperature treatment was further examined by qRT-PCR. The qRT-PCR results were consistent with the FPKM values, showing a progressive increase in *PiLUX* expression with prolonged cold treatment (Figure 2a). The results show that the expression level of this gene in leaves gradually increased as the duration of low-temperature treatment prolonged, indicating that *PiLUX* expression exhibits a cold-induced accumulation trend and that this gene is involved in the cold stress response of *P. indica*.

#### 3.2.2. Analysis of Correlation Between PiLUX Expression and Physiological and Biochemical Parameters

To elucidate the physiological function of *PiLUX*, we measured the relative electrolyte leakage (EL), Superoxide Dismutase (SOD), Peroxidase (POD), and Catalase (CAT) enzyme activities, as well as MDA and soluble sugar content, in leaves and performed a correlation analysis with *PiLUX* expression levels. The heatmap (Figure 2b) shows that *PiLUX* expression is significantly positively correlated with EL, SOD, POD, and CAT enzyme activities and soluble sugar content, whilst it is negatively correlated with MDA. This suggests that *PiLUX* may synergistically contribute to the adaptive response of *P. indica* to low temperatures by integrating multiple pathways, including antioxidant defense, osmotic regulation, and maintenance of membrane stability.

#### 3.2.3. Analysis of the Conserved Domains and Phylogenetic Relationships of PiLUX Proteins

Using the cDNA from *P. indica* leaves as a template and gene-specific primers designed from annotated unigene sequences, we amplified the full-length coding sequence (CDS) of *PiLUX*. Agarose gel electrophoresis revealed a single, bright band above the 1000 bp molecular weight marker, consistent with the expected size of the 1050 bp amplification product (Figure S4). Sanger sequencing of the PCR product showed a perfect match (100% identity) to the unigene sequence derived from the transcriptome, confirming successful cloning of the complete open reading frame (ORF) of *PiLUX*.

Bioinformatics analysis predicted that PiLUX encodes a 349-amino-acid polypeptide, with a theoretical molecular weight of 37.75 kDa and a theoretical isoelectric point (pI) of 5.82. The protein’s instability index (II) is 47.92, its aliphatic amino acid index is 58.17, and its global average hydrophilicity (GRAVY) is –0.718, indicating that the protein is relatively unstable and hydrophilic. A BLASTp search of the NCBI Non-Redundant Protein Database (NCBI BLAST+ v2.12.0) revealed that PiLUX exhibits high sequence similarity to the LUX homologs from other plant species. Multiple sequence alignment further identified the presence of a G2-like conserved domain and a MYB-like DNA-binding domain in the inferred amino acid sequence, located at residues 150–360 and 163–216, respectively (Figure 2c). A phylogenetic tree was constructed using the agglomerative method, showing that the overall amino acid sequence similarity between PiLUX and other plant LUX homologs is approximately 65%; however, it does not form a closely related clade with any LUX homologs with high support, indicating that it occupies a relatively isolated position within the phylogenetic tree (Figure 2d).

### 3.3. Subcellular Localization Results

**Figure 3:**
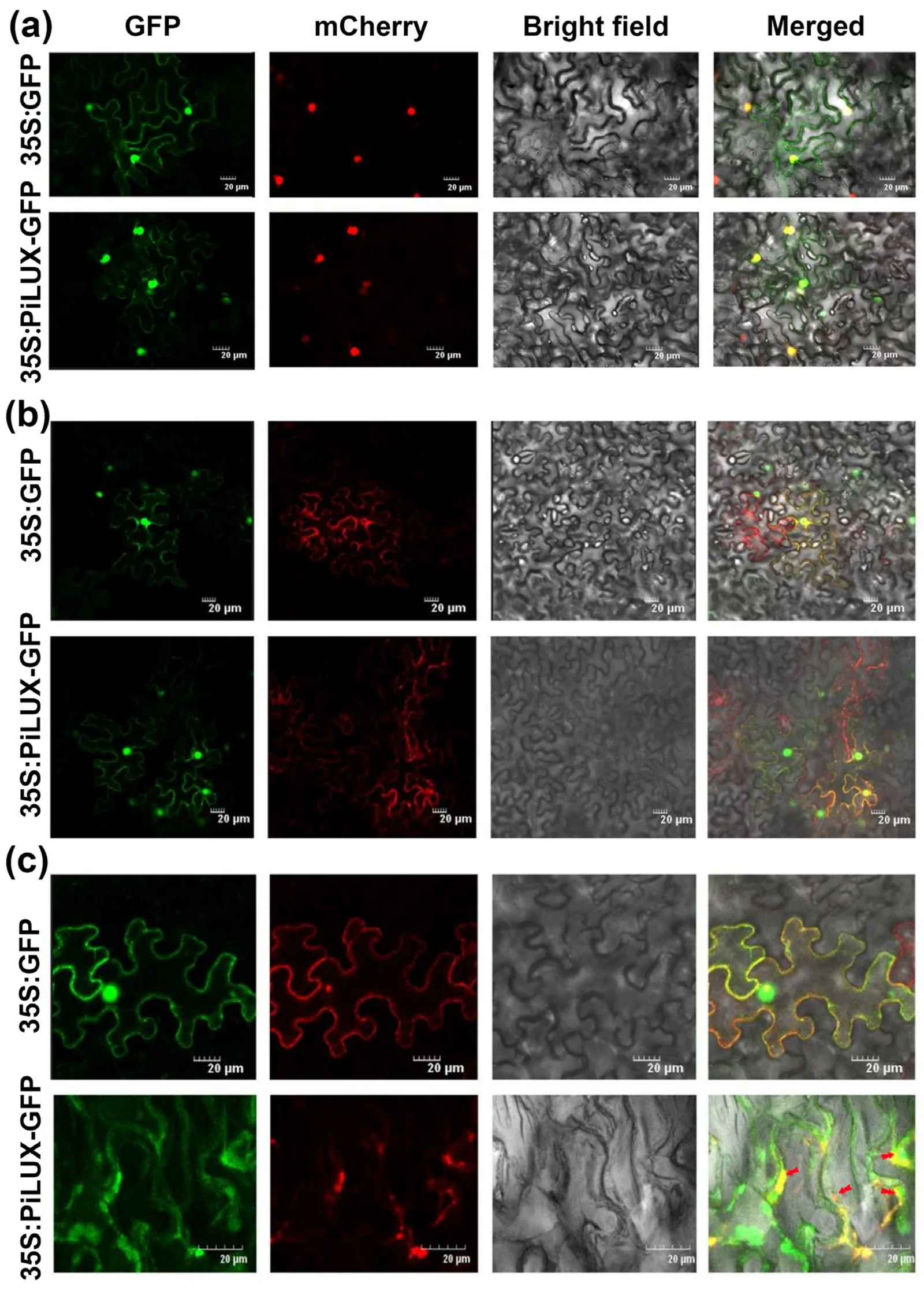
PiLUX subcellular localization results. (**a**) Co-injection of an empty vector (35S: GFP) or the transforming vector 35S: LUX-GFP with the nuclear localization signal (NLS) gene into tobacco leaves. (**b**) Co-injection of an empty vector (35S: GFP) or the transforming vector 35S: LUX-GFP with the membrane marker gene PIP2 into tobacco leaves. (**c**) Tobacco leaves after plasmolysis separation. From left to right: confocal microscopic images of epidermal cells obtained for observation in the green fluorescence field (GFP), red fluorescence field (mCherry), bright field, and superimposed field; arrows represent the location of cell plasma wall separation. Scale bar = 20 µm.

To determine its subcellular localization, PiLUX was cloned into the BG-Plant-GFP vector, driven by the CaMV 35S promoter, with a 35S; the GFP empty vector served as the negative control. Observation of tobacco leaves using fluorescence confocal microscopy revealed widespread GFP signal in the epidermal cells (cytoplasm and nucleus) of the control vector-transformed group; PiLUX was widely distributed within these cells, including in the nucleus, and colocalized with the nuclear localization signal (NLS) gene (Figure 3a). To determine the specific site of action of PiLUX, co-injection with the membrane marker gene plasma membrane intrinsic protein 2 (PIP2) was subsequently performed.

As the cell membrane and cell wall are in direct contact under normal conditions, it is not possible to distinguish the site of expression (Figure 3a). Consequently, we performed a plasmolysis experiment on tobacco leaf cells to induce a state of plasmolysis (Figure 3b). The results show that, following plasmolysis, PIP2 was specifically expressed in the cell membrane, producing red fluorescence under mCherry illumination; PiLUX was expressed in the nucleus, cell membrane, and cell wall, producing green fluorescence under GFP illumination; when the images were overlaid, PiLUX and PIP2 were found to be co-expressed in the cell membrane, resulting in yellow fluorescence. These results indicate that PiLUX is widely distributed within epidermal cells and is expressed not only in the cell membrane but also in the cell wall, emitting distinct green fluorescence, as observed in the overlaid image (Figure 3c).

### 3.4. Effects of PiLUX Overexpression on A. thaliana’s Growth, Development, and Tolerance to Low Temperatures

#### 3.4.1. Generation of *A. thaliana* Lines Overexpressing PiLUX

**Figure 4:**
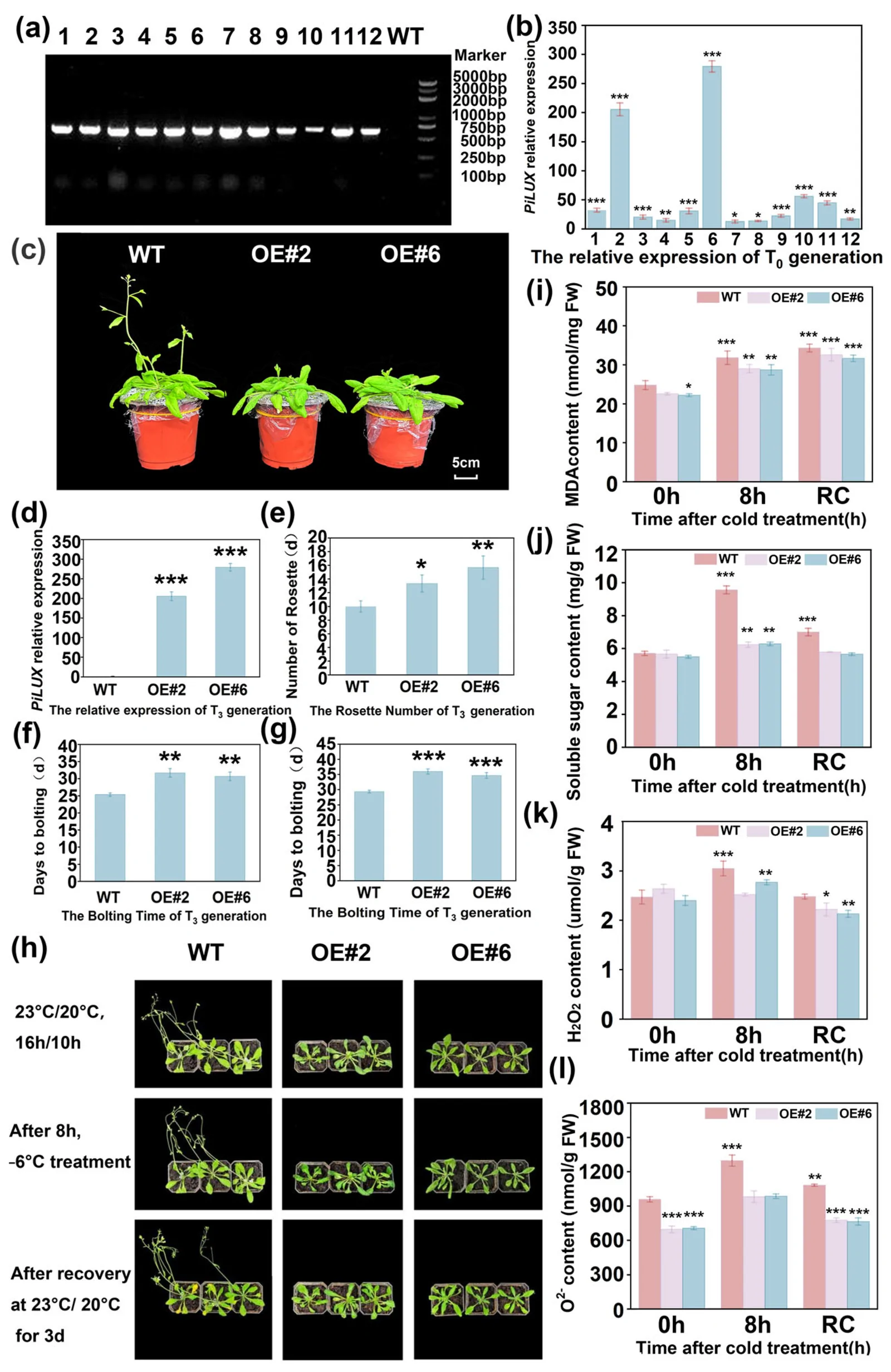
Phenotypic characterization, growth regulation, and analysis of physiological response to low temperatures in *PiLUX*-overexpressing *A. thaliana* lines. (**a**) PCR electrophoresis gel showing the identification of transgenic-positive plants in the T_0_ generation; (**b**) relative expression levels of the *PiLUX* gene in transgenic plants; (**c**) phenotypes of WT and *PiLUX*-overexpressing lines at 30 days after sowing; (**d**) *PiLUX* expression levels in WT and *PiLUX*-overexpressing *A. thaliana* lines; (**e**) number of rosette leaves in WT and *PiLUX*-overexpressing lines; (**f**) time to bolting in WT and *PiLUX*-overexpressing lines; (**g**) time to flowering in WT and PiLUX-overexpressing lines; (**h**) phenotypic symptoms of WT and *PiLUX*-overexpressing transgenic *A. thaliana* following low-temperature stress; (**i**) MDA content; (**j**) soluble sugar content; (**k**) H_2_O_2_ content; (**l**) O^2−^ content. In (**b**,**i**–**l**), error bars represent ± SD (*n* = 3); in (**i**–**l**), OE#2 and OE#6 represent two independent T_3_-generation *PiLUX*-overexpressing transgenic lines; in (**d**–**g**), error bars represent ± SD (*n* = 27). *** *p* < 0.001, ** *p* < 0.01, and * *p* < 0.05.

Kanamycin resistance screening was performed on T_1_-generation *A. thaliana* seedlings; candidate lines were randomly selected from the surviving plants and identified via PCR using specific primers (Table S1). A total of 12 independent transgenic lines stably expressing *PiLUX* were obtained (Figure 4a). Based on qRT-PCR analysis, lines #2 and #6, which exhibited the highest expression levels, were selected for further purification (Figure 4b). Following successive kanamycin resistance selection up to the T_3_ generation, homozygous overexpression lines were obtained for subsequent phenotypic and physiological analyses.

#### 3.4.2. Phenotypic Analysis of PiLUX-Overexpressing *A. thaliana*

Compared with the wild-type (WT), the T_3_-generation *PiLUX*-overexpressing lines exhibited a marked delay in flowering. At 30 days of growth, WT plants had already bolted and entered the flowering stage, whilst the *PiLUX*-overexpressing lines #2 and #6 (OE#2 and OE#6) were still in the early stages of bolting or had not yet bolted (Figure 4c). qRT-PCR validation indicated that PiLUX was expressed at extremely high levels in both OE#2 and OE#6 (Figure 4d). In terms of vegetative growth, the number of rosette leaves in OE#2 and OE#6 was significantly higher than that in the WT, by 3.3 and 5.6 leaves, respectively (Figure 4e). Regarding the transition to reproductive growth, the average time to bolting in OE#2 and OE#6 was delayed by 6.3 days and 5.3 days, respectively, compared with the WT (Figure 4f), whilst the average time to flowering was delayed by 6.6 days and 5.3 days, respectively (Figure 4g).

#### 3.4.3. Evaluation of Low-Temperature Tolerance in PiLUX-Overexpressing *A. thaliana*

The T_3_-generation OE#2, OE#6, and WT plants, which had been grown for 30 days, were subjected to an 8-h low-temperature treatment at −6 °C, followed by 3 days of recovery under normal conditions. Following the treatment, it was observed that the leaves of all lines exhibited some degree of wrinkling; after 3 days of recovery, the WT leaves displayed severe symptoms of yellowing and wilting, whilst the OE#2 and OE#6 plants had largely recovered to normal, with only localized wilting at the leaf tips (Figure 4h). These phenotypic differences indicate that *PiLUX* overexpression enhances the tolerance of transgenic *A. thaliana* to low temperatures.

To investigate the physiological mechanisms by which *PiLUX* overexpression enhances tolerance to low temperatures, the levels of MDA, soluble sugars, H_2_O_2_, and O^2−^ in the leaves of WT, OE#2, and OE#6 plants were measured prior to low-temperature treatment (control), 8 h after low-temperature treatment at −6 °C, and 3 days after recovery.

Prior to the low-temperature treatment, the MDA levels in OE#2 and OE#6 were both lower than that in the WT. After 8 h of low-temperature treatment, the MDA levels in all three strains increased significantly. After 3 days of recovery, the MDA levels in all three strains had decreased but had not yet returned to the pre-treatment levels; however, the MDA levels in OE#2 and OE#6 remained lower than that in the WT, indicating that *PiLUX* overexpression effectively mitigates the extent of low-temperature-induced membrane lipid peroxidation (Figure 4i).

Regarding soluble sugar content, there were no significant differences between the WT, OE#2, and OE#6 prior to the low-temperature treatment. Following 8 h of low-temperature treatment, the soluble sugar content increased significantly by 1.68-fold, 1.10-fold, and 1.14-fold, respectively, in the WT, OE#2, and OE#6 relative to their pre-treatment levels. After 3 days of recovery, the soluble sugar content in OE#2 and OE#6 had largely returned to the pre-treatment levels, being at 1.02-fold and 1.03-fold of the pre-treatment levels, respectively, whilst the soluble sugar content in the WT remained at a higher level, being at 1.23-fold of the pre-treatment level (Figure 4j). These results indicate that the OE lines experienced a milder degree of osmotic stress compared with the WT and possessed a stronger recovery capacity.

Regarding H_2_O_2_ content, there were no significant differences between the WT, OE#2, and OE#6 prior to the low-temperature treatment. Following 8 h of low-temperature treatment, the H_2_O_2_ content in the WT showed a significant increase to 1.24 times the pre-treatment level, whilst the H_2_O_2_ content in OE#2 and OE#6 were 0.95 times and 1.15 times the pre-treatment levels, respectively. After 3 days of recovery, the H_2_O_2_ content in the WT had largely returned to the pre-treatment level, whilst the H_2_O_2_ content in OE#2 and OE#6 had decreased further to 0.84-fold and 0.89-fold of the pre-treatment levels, respectively (Figure 4k). These results indicate that the *PiLUX*-overexpressing lines possess a more efficient ROS scavenging capacity under low-temperature stress and during the subsequent recovery process, which mitigates oxidative stress damage.

Prior to the low-temperature treatment, the O^2−^ levels in OE#2 and OE#6 were significantly lower than that in the wild-type (WT). Following 8 h of low-temperature treatment, the O^2−^ levels increased significantly in all three lines, reaching 1.35-fold, 1.41-fold, and 1.40-fold of their pre-treatment levels, respectively, for the WT, OE#2, and OE#6. After 3 days of recovery, the O^2−^ levels in OE#2 and OE#6 had largely returned to the pre-treatment levels, whilst the O^2^ level in the WT remained significantly higher (Figure 4l). Throughout the low-temperature stress and recovery process, the levels in both *PiLUX*-overexpressing lines remained consistently lower than those in the WT, suggesting that *PiLUX* overexpression may maintain a lower level of O^2−^ accumulation, thereby mitigating the oxidative damage caused by cold stress.

### 3.5. PiLUX Gene Knockdown Reduces the Cold Tolerance of P. indica

#### 3.5.1. Generation of PiLUX-Knockdown Plants

**Figure 5:**
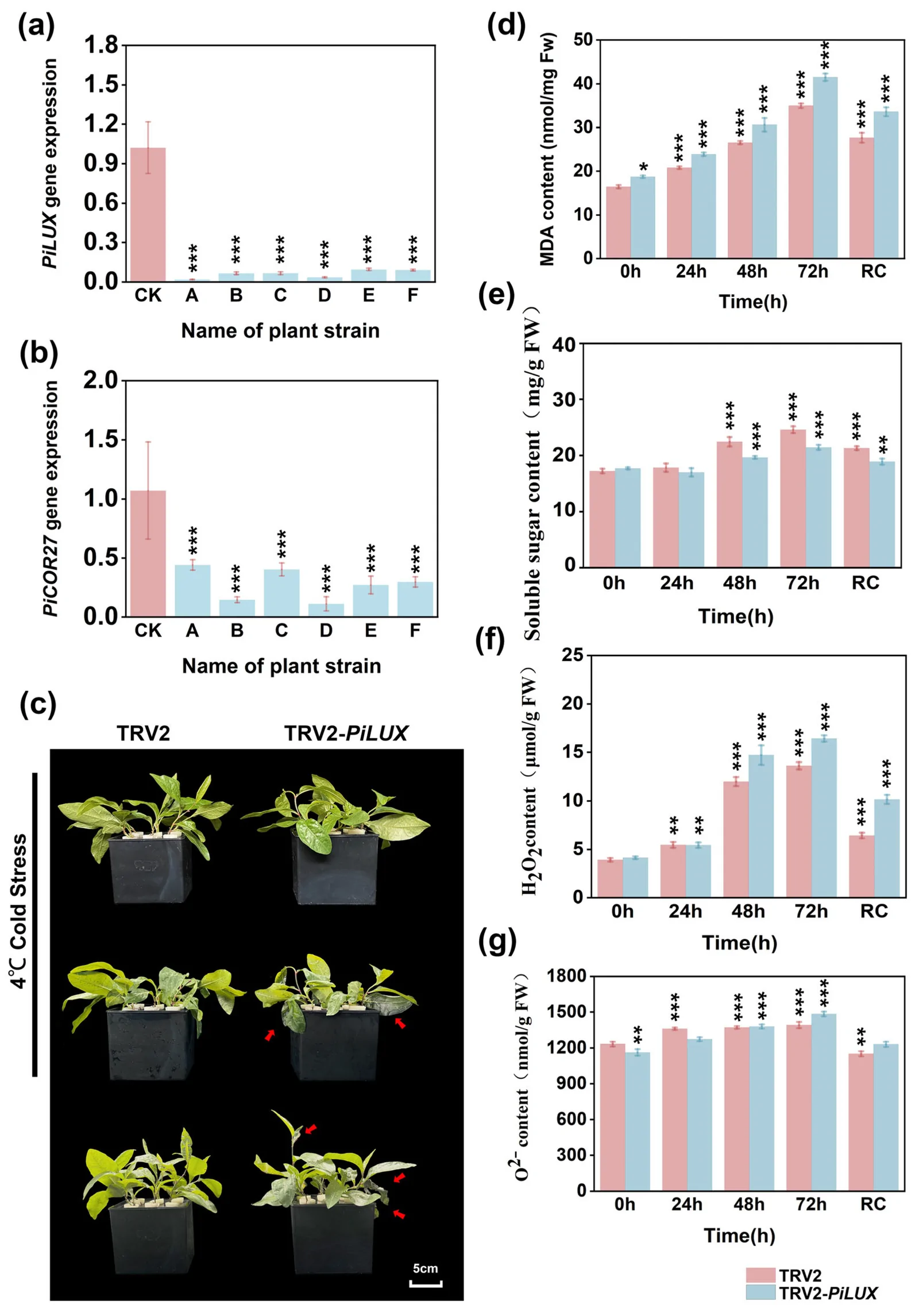
Effects of VIGS-mediated knockdown of *PiLUX* on low-temperature tolerance in *P. indica*. (**a**) Assessment of knockdown efficiency in the TRV2-*PiLUX* strain. (**b**) Expression levels of the *PiCOR27* gene following PiLUX knockdown. (**c**) Phenotype of *P. indica*; from top to bottom, the three time points represent 0 h, 72 h, and 72 h after recovery from low-temperature stress, respectively; from left to right, the control TRV2 and the VIGS strain TRV2-*PiLUX* are shown, respectively. Scale bars = 5 cm; red arrows indicate irreversible damage to *P. indica* leaves. (**d**) MDA content; (**e**) soluble sugar content; (**f**) H_2_O_2_ content; (**g**) O^2−^ content. Data are presented as mean ± SD (*n* = 3). Asterisks indicate significant differences between TRV2 and TRV2-*PiLUX* plants (* *p* < 0.05, ** *p* < 0.01, *** *p* < 0.001). TRV2, tobacco rattle virus vector 2; VIGS, virus-induced gene silencing; RC, recovery.

Six *P. indica PiLUX*-knockdown plants were identified via qRT-PCR and labeled A, B, C, D, E, and F. The knockdown efficiencies were 98.15%, 93.37%, 93.32%, 96.44%, 90.45%, and 90.95%, respectively, with an average knockdown efficiency of 93.78%, indicating a high level of knockdown efficiency (Figure 5a). To investigate the role of *PiLUX* in the cold-tolerance pathway, the expression levels of the potential downstream cold-tolerance gene *PiCOR27* were analyzed. The results indicate that following *PiLUX* knockdown, the expression of the *PiCOR27* gene in the low-temperature-responsive pathway was significantly downregulated (Figure 5b). The *COR27* gene has been shown in previous studies to be regulated by *LUX* and belongs to the cold-tolerance gene family. This suggests that *PiLUX* knockdown may have reduced the cold tolerance of *P. indica*.

#### 3.5.2. Phenotypic Observations Under Low-Temperature Stress

Phenotypic observations revealed that during low-temperature treatment, TRV2 plants gradually exhibited mild wilting symptoms, whilst TRV2-*PiLUX* plants displayed more severe wilting, curling, and yellowing under the same conditions. After 72 h of recovery, the TRV2 plants had largely returned to their pre-treatment state, showing only minor damage; in contrast, the TRV2-*PiLUX* plants were unable to recover fully, with some leaves exhibiting irreversible damage (Figure 5c).

#### 3.5.3. Measurement of Physiological Parameters Under Low-Temperature Stress

To investigate the physiological mechanism by which *PiLUX* knockdown reduces cold tolerance, the MDA, soluble sugar, H_2_O_2_, and O^2−^ levels were measured in TRV2 and TRV2-*PiLUX* plant leaves at five time points: prior to the low-temperature treatment (0 h); at 24, 48, and 72 h after treatment; and 72 h after recovery (RC).

Prior to the low-temperature treatment, the MDA content in TRV2-*PiLUX* was significantly higher than that in TRV2. During the low-temperature treatment, the MDA levels in both lines showed a continuous upward trend as cold stress duration increased. After 72 h of recovery, the MDA levels in both TRV2 and TRV2-*PiLUX* decreased, but did not return to their pre-treatment levels, indicating that low temperatures cause irreversible cellular damage. However, the MDA content in TRV2-*PiLUX* remained consistently higher than that in TRV2, suggesting that *PiLUX* knockdown exacerbates the extent of membrane lipid peroxidation induced by low temperatures (Figure 5d).

Regarding soluble sugar content, there were no significant differences between TRV2 and TRV2-*PiLUX* prior to the low-temperature treatment and 24 h after the treatment. Subsequently, at 48 and 72 h, the soluble sugar content in TRV2 gradually increased, whilst in TRV2-*PiLUX*, it peaked at 72 h. After 72 h of recovery, the soluble sugar contents of TRV2 and TRV2-*PiLUX* were 1.24-fold and 1.06-fold, respectively, of their pre-treatment levels (Figure 5d). These results indicate that the TRV2 strain accumulated more soluble sugars during the later stages of low-temperature stress as well as during the recovery phase.

Regarding the H_2_O_2_ content, there was no significant difference between TRV2 and TRV2-*PiLUX* prior to the low-temperature treatment. Following 48 h of low-temperature treatment, the levels in both strains rose significantly, peaking at 72 h, with the increase in TRV2-*PiLUX* being significantly higher than that in TRV2. Following a 72-h recovery period, the levels in both lines decreased, reaching 1.64- and 2.45-fold of their pre-treatment levels, respectively (Figure 5e). These results indicate that the *PiLUX*-knockdown line exhibits relatively higher H_2_O_2_ accumulation under low-temperature stress, resulting in more severe oxidative damage.

Prior to the low-temperature treatment, the O^2−^ levels in TRV2-*PiLUX* were significantly lower than those in TRV2. After 24 h of low-temperature treatment, the O^2−^ levels in both lines increased; by 72 h, the O^2−^ levels in both lines had reached their respective peaks. After 72 h of recovery, the O^2−^ levels in both TRV2 and TRV2-*PiLUX* decreased (Figure 5f). Throughout the low-temperature stress and recovery process, the O^2−^ content in TRV2-*PiLUX* was higher than that in TRV2, suggesting that *PiLUX* knockdown led to increased O^2−^ accumulation, thereby exacerbating oxidative stress damage.

## 4. Discussion

### 4.1. PiLUX Is a Circadian Gene That Confers Cold Tolerance to P. indica

Recent studies have shown that plant responses to low temperatures are not merely passive stress strategies but are also actively regulated by endogenous circadian systems. This regulatory mechanism enables plants to anticipate and adapt to periodic fluctuations in diurnal environmental factors [42,43,44]. This view is consistent with the findings of the present study; the KEGG enrichment analysis results show that the ‘circadian rhythm–plant’ pathway is among the most significantly enriched pathways in the transcriptome of *P. indica* under low temperatures, suggesting that low-temperature stress triggers downstream responses of circadian rhythm-related genes. Although this study aimed to capture transcriptional responses in the early stages of low-temperature stress, rather than to systematically characterize the phasic dynamics of circadian rhythms, the significant enrichment of this pathway provides a clear entry point for further investigation into the cross-regulation between the circadian clock and low-temperature signaling. Notably, we identified the expression of the core circadian rhythm gene *PiLUX* as being induced by low temperatures, and its transcriptional levels were found to be significantly and positively correlated with multiple cold-tolerance-related physiological indicators.

Under low temperatures, circadian rhythm-related transcription factors form a complex regulatory network governing *CBF* expression: *PRRs* negatively regulate *CBF* expression and cold tolerance [45]; *RVE4* and *RVE8*, in turn, migrate from the cytoplasm to the nucleus in response to low temperatures, directly inducing the expression of *CBF2* and *CBF3* [43]. The role of *CCA1* and *LHY* in regulating *CBFs* has been controversial—earlier studies suggested that they directly promote the low-temperature-induced expression of the three *CBF* genes [45,46,47], whereas recent studies indicated that they act as transcriptional repressors [43]. This discrepancy may stem from antagonistic regulation between the two *CCA1* splicing variants—*CCA1α* and *CCA1β*—as well as the rapid degradation of CCA1/LHY proteins at low temperatures [48]. Furthermore, *CCA1* positively regulates low-temperature tolerance during nocturnal cooling by modulating the expression of *COR27* and *COR28* [49]. These findings indicate that the entire circadian system is involved in the perception and transmission of low-temperature signals.

Previous studies have shown that JA signaling can enhance cold tolerance in plants and is closely associated with the classical cold signaling cascade *ICE1-CBFs-COR* [50,51]. In *A. thaliana*, the transcriptional activity of ICE1 is suppressed by its physical interaction with JAZ1; in tea plants, the CsLUX protein not only attenuates the inhibition of CsICE1 by CsJAZ1 but may also directly bind to the *CsJAZ1* promoter to regulate its expression, thereby participating in the feedback regulation of JA signaling at the transcriptional level [35]. Notably, *CBF1*, as a core transcription factor in low-temperature signaling, has been shown to directly upregulate *LUX* expression [32,49], and *LUX* subsequently positively regulates plant cold tolerance.

**Figure 6:**
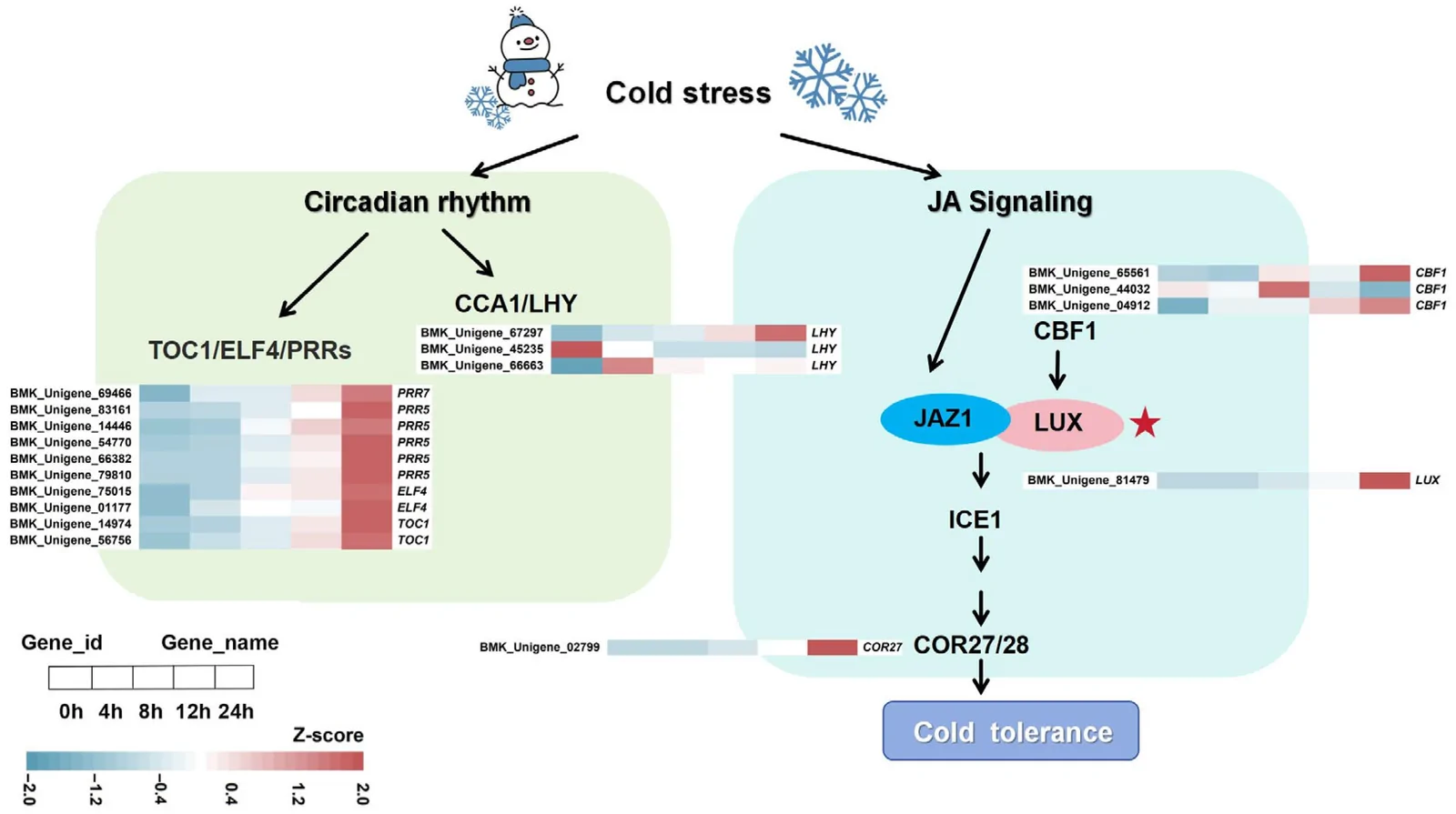
A hypothetical working model of signaling pathway convergence in *P. indica* under low temperatures, with *PiLUX* as a candidate node. Note: In this model, we highlight several relevant genes that exhibit differential expression under low temperatures, with |log_2_FC| ≥ 1.0. The red asterisk indicates that *LUX*, the core gene in this study, is of particular interest; it exhibits sustained upregulation under low-temperature treatment. The heatmap displays the relative expression levels (Z-score, normalized) of each gene in terms of FPKM at 0 h (h), 4 h, 8 h, 12 h, and 24 h of low-temperature treatment. Arrows indicate previously reported direct regulatory relationships in other plant species or proposed relationships that require experimental validation in *P. indica*. The model is based on transcriptomic correlation and expression timing, and does not by itself demonstrate direct regulation. The asterisk indicates the target gene *PiLUX*.

By integrating the transcriptomic data from this study with previously established molecular frameworks of plant temperature responses [10,35,52], we constructed a hypothetical working model in which *PiLUX* is a candidate node connecting circadian regulation and low-temperature signaling (Figure 6). This model is based on transcriptomic correlation and expression timing, and the proposed regulatory relationships require further experimental validation. This model is based on current understanding of the crosstalk between low-temperature signaling, circadian rhythms, and the hormone JA. Our transcriptomic data show that homologs of the aforementioned pathway nodes (*CCA1/LHY*, *PRRs*, *TOC1*, *CBF1*, *COR27/COR28*, etc.) exhibit dynamic expression responses to low-temperature treatment. Interestingly, *PiCBF1*, *PiLUX*, and *PiCOR27* all show significant and sustained upregulation in *P. indica* in response to low-temperature treatment; chronologically, *PiCBF1* accumulates continuously before *PiLUX*, and *PiLUX* accumulates continuously before *PiCOR27*. This regulatory pattern is consistent with the findings in tea plants that *CsCBF1* activates *CsLUX* and that overexpression of *CsLUX* promotes the expression of downstream *COR* genes [35]. This similarity suggests that a *CBF1*-*LUX*-*CORs* module may also operate in *P. indica*, but direct evidence for such a module in this species is still lacking. Direct regulation of downstream targets by *PiLUX*, and its possible placement within the CBF pathway, require further experimental evidence at the protein level.

To further assess the reliability of the transcriptome data, the expression of six key genes in the proposed model, *PRR5*, *CBF1*, *LHY, COR27*, *TOC1*, and *ELF4,* was examined by qRT-PCR. The results were largely consistent with the RNA-seq data (Supplementary Figure S5). The primers used for qRT-PCR detection are shown in Table S1.

### 4.2. PiLUX Synergistically Enhances Cold Tolerance Through Multiple Physiological Pathways and Delays Plant Flowering

The physiological data from this study indicate that *PiLUX* overexpression synergistically enhances cold tolerance in transgenic *A. thaliana* through multiple pathways, including reducing membrane lipid peroxidation, maintaining osmotic homeostasis, and enhancing ROS scavenging capacity. Notably, under ambient-temperature conditions, the MDA and O^2−^ levels in the *PiLUX*-overexpressing lines were found to be already significantly lower than those in the wild-type, suggesting that *PiLUX*-overexpressing plants may have established a more efficient redox buffering system even before the onset of cold stress, giving these transgenic plants a pre-adaptive advantage when low temperatures occur. This phenomenon is similar to that reported by Li et al. [49] for *COR27*-overexpressing lines, which also exhibited lower ROS levels under non-stress conditions.

However, this study also revealed a potential adaptive cost: *PiLUX* overexpression significantly delayed flowering. In *A. thaliana*, *LUX*, as a core component of the EC, can directly or indirectly regulate the transcription of the flowering integrator factor *FT*. Furthermore, heterologous overexpression of *PiLUX* in *A. thaliana* may suppress the expression of endogenous *AtFT* through conserved regulatory mechanisms, thereby delaying the flowering transition [33,53,54]. This suggests a trade-off between enhanced cold tolerance and reproductive development—plants accumulate more carbon reserves and stress-responsive metabolites by prolonging the vegetative growth period, thereby enhancing their tolerance to low temperatures [55,56]. This trade-off is ecologically rational: delaying flowering under low temperatures protects reproductive organs from frost damage, thereby increasing reproductive success [57]. However, for species such as *P. indica*, whose primary economic traits are its ornamental value and medicinal components, how to balance these plants’ cold tolerance, flowering time, and accumulation of secondary metabolites for genetic improvement will be a key scientific issue requiring focused attention in future research.

## 5. Conclusions

In this study, the core circadian rhythm gene *PiLUX* in *P. indica* was characterized and cloned, and its key regulatory role in the plant’s cold-stress response was systematically elucidated. Transcriptomic analysis and physiological experiments indicated that low temperatures significantly induce the expression of *PiLUX*, and its expression level is positively correlated with the plant’s ROS scavenging capacity, membrane stability, and osmotic regulatory capacity. Heterologous overexpression of *PiLUX* enhances cold tolerance in transgenic *A. thaliana* but simultaneously delays flowering; conversely, VIGS-mediated silencing of *PiLUX* reduces cold tolerance in *P. indica* and exacerbates oxidative damage. Based on the transcriptomic data and the functional validation performed here, we propose a hypothetical working model in which *PiLUX* is a candidate node connecting circadian regulation and low-temperature signaling. In this model, *PiLUX* may contribute to cold tolerance by influencing downstream low-temperature-responsive genes; however, direct regulation of downstream targets by *PiLUX* and its possible placement within the CBF pathway require further protein-level experimental evidence. Direct validation of this proposed regulatory relationship is an important direction for future work. This study provides new insights into the adaptation strategies of thermophilic plants under low-temperature stress. It also offers a theoretical basis and genetic resources for cold-tolerance breeding in *P. indica*.

## Data Availability

The original contributions presented in this study are included in the article/supplementary material. Further inquiries can be directed to the corresponding author.

## Supplementary Materials

The following supporting information can be downloaded at https://www.mdpi.com/article/10.3390/plants15193032/s1, Figure S1: Vector map of BG-Plant-GFP; Figure S2: Sequencing of PiLUX inserted into the BG-Plant-GFP vector; Figure S3: Sequencing of PiLUX inserted into the TRV-RNA2 vector; Figure S4: PCR amplification results of PiLUX; Figure S5: RT-qPCR validation of the expression patterns of key genes in the proposed low-temperature regulatory model of P. indica; Table S1: List of primers used in this study.

## Abbreviations

The following abbreviations are used in this manuscript:

VIGS	Virus-induced gene silencing
*CBF*	C-repeat binding factor
At	*Arabidopsis thaliana*
*Pi*	*Plumbago indica* L.
ROS	Reactive oxygen species
*COR*	Cold-regulated genes
OE	Overexpression
WT	Wild-type
Cs	*Camellia sinensis*
*Os*	*Oryza sativa*
*Pa*	*Plumbago auriculata* Lam.
qRT-PCR	Quantitative real-time PCR
