# Supplementary material for: The Core Circadian Clock Gene PiLUX, a MYB-Family Transcription Factor, Enhances Cold Tolerance in Plumbago indica L
Source: Plants (Basel). 2026 Oct 3;15(19):3032. doi: 10.3390/plants15193032 (PMC13667146; doi:10.3390/plants15193032)
Supplement: Supplementary file 1 [file plants-15-03032-s001.zip › plants-4579076-supplementary.pdf]

Table S1. List of Primers used in this study

NO.	Purpose	Primers (5′→3′)
1	Identification of positive Arabidopsis transformants-F	CTTACTTAACCAACAACTCCTCCTCTT
	Identification of positive Arabidopsis transformants-R	ACTTTCACCCAACACTGTCCCT
2	qPCR for *PiLUX* in positive Arabidopsis transformants-F	GAACACTCTTGCCACCCTCA
	qPCR for *PiLUX* in positive Arabidopsis transformants-R	CGCATCTTCCTCGAATCGGA
3	*PiLUX*-VIGS construct-F	CCGGAATTCATGGGAACAATGGACAAGTGGGAAT
	PiLUX-VIGS construct-R	CGGGGTACCTCACCCGCTGAAAGATGG
4	*PiLUX*-qPCR-F	GCCGCCTTCCCGCACAAC
	PiLUX-qPCR-R	ACCGCTCGTCGTCGTCGTC
5	*PiCOR27*-qPCR-F	AGTTGGCAAGGAACTTGGGT
	PiCOR27-qPCR-R	GCCTGCACAATCTCACATCG
6	*PiPRR5*-qPCR-F	GCTGGGTTTGGCCCTATCTT
	PiPRR5-qPCR-R	TCCCCACATCAGAACGCAAA
7	*PiCBF1*-qPCR-F	CGCCGGATTCATGTCCGATA
	PiCBF1-qPCR-R	CGCCTCCTTGTGATTCCTCA
8	*PiLHY*-qPCR-F	GGATGGGGATGTCCTGCAAA
	PiLHY-qPCR-R	ACGATTGAGACTCGGCTTCC
9	*PiTOC1*-qPCR-F	GAGCTGCTGTGCAAGTGTTC
	PiTOC1-qPCR-R	TGGTCCTTCCGCGTTAAGAG
10	*PiELF4*-qPCR-F	AGGGATCTCATCCAGCAGGT
	PiELF4-qPCR-R	GAAGCTACGGCGGAGATGTT

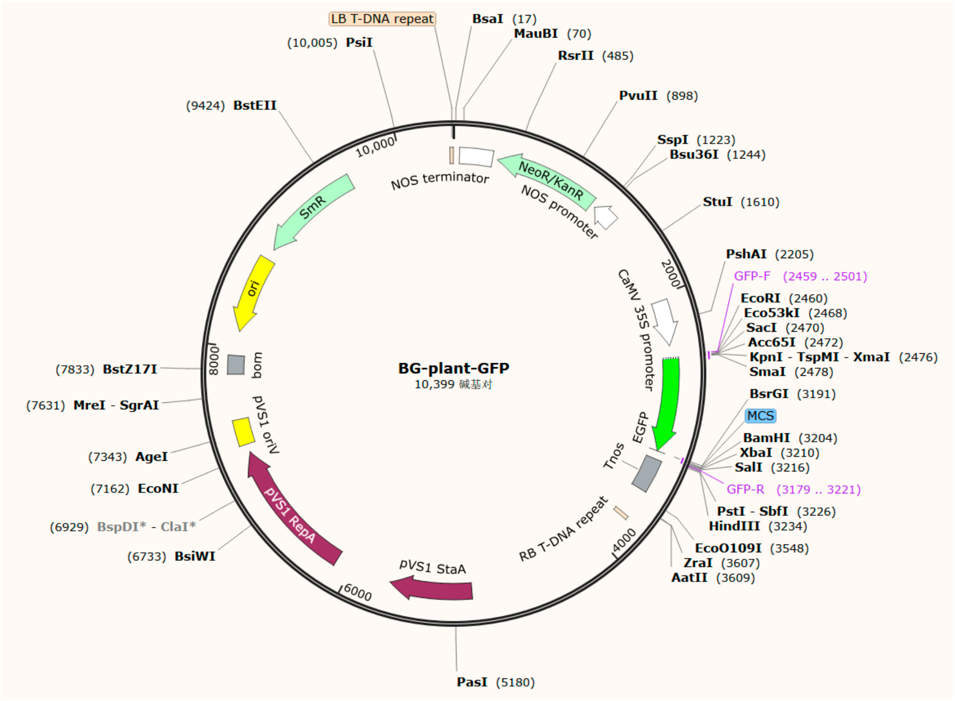

Supplementary Figure S1. BG-Plant-GFP Carrier map

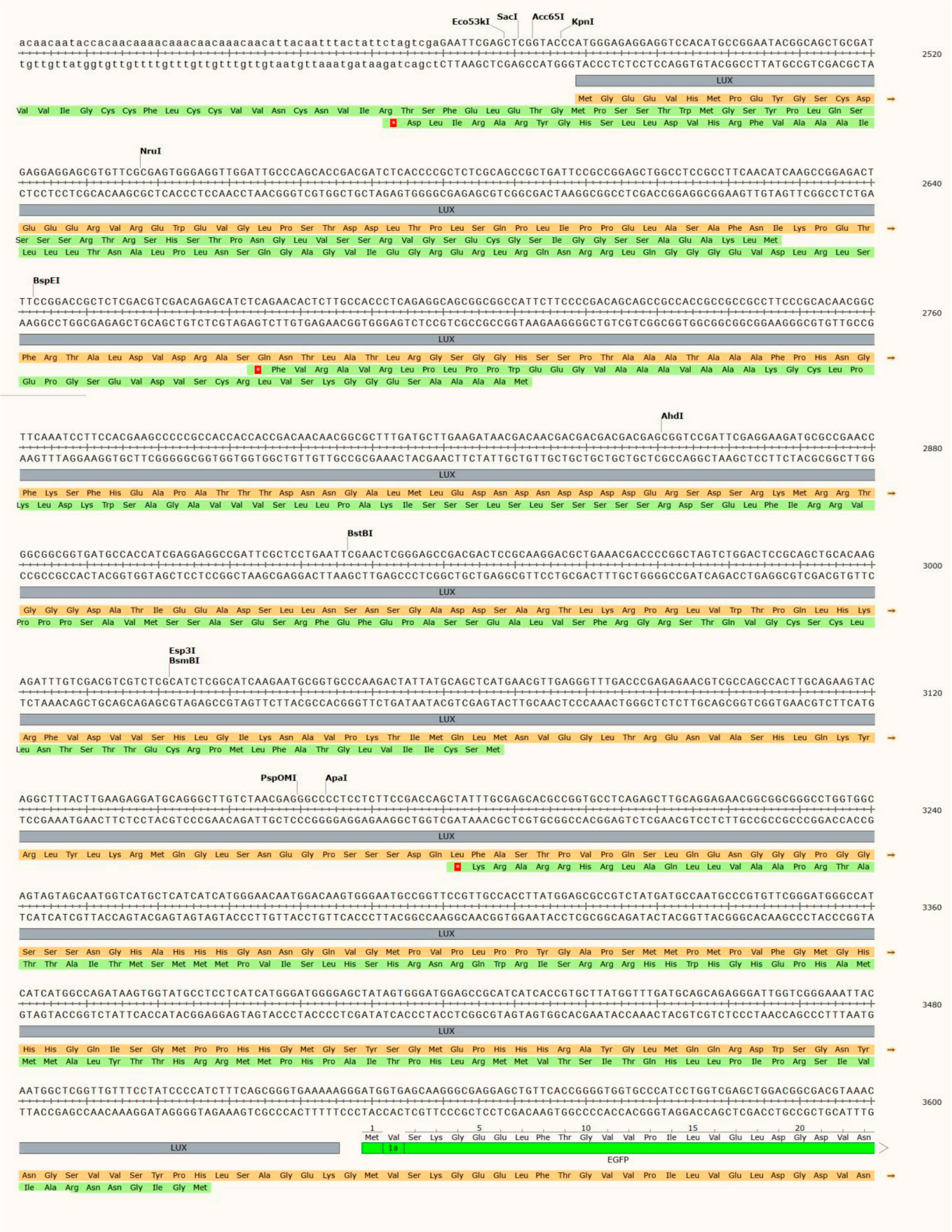

Supplementary Figure S2. PiLUX was sequenced by insertion of BG-Plant-GFP vector

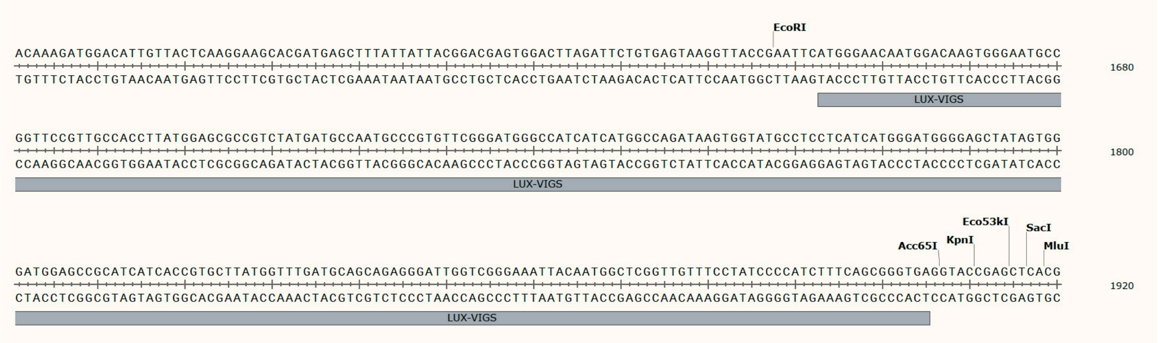

Supplementary Figure S3. ***PiLUX*** **was sequenced by insertion of TRV-RNA2 vector**

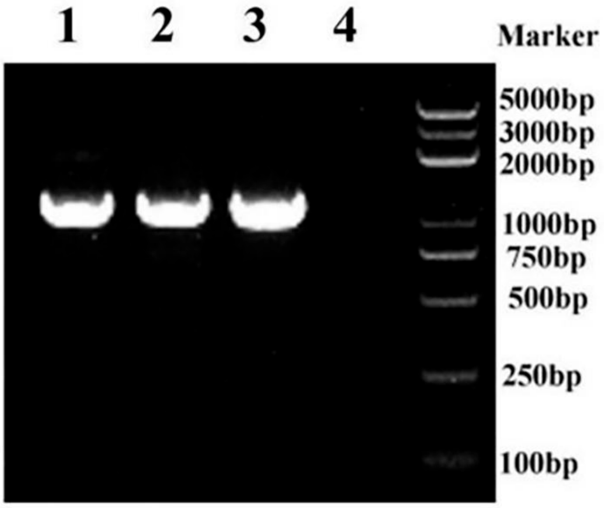

Supplementary Figure S4. PCR amplification results of ***PiLUX***

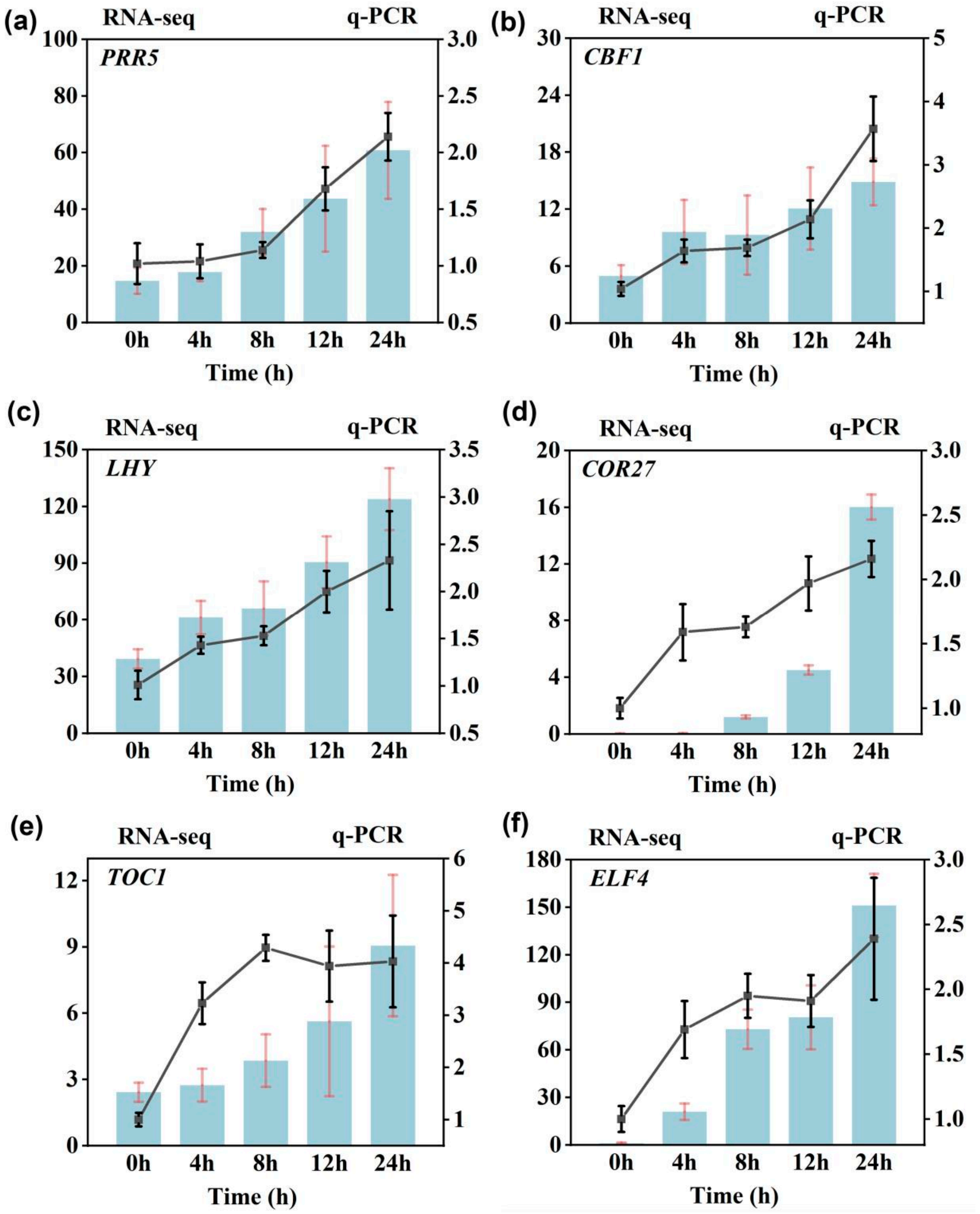

Supplementary Figure S5. RT-qPCR validation of the expression patterns of key genes in the proposed low-temperature regulatory model of *P. indica*

(a) *PRR5*; (b) *CBF1*; (c) *LHY*; (d) *COR27*; (e) *TOC1*; (f) *ELF4*. For each panel, the X-axis represents the time of low-temperature treatment (0, 4, 8, 12, and 24 hours), the left Y-axis displays the corresponding RNA-seq expression data (blue histogram), and the right Y-axis shows the relative gene expression level determined by RT-qPCR (black dash line). Data are presented as mean ± SD (n = 9).
